# Identification of Gingivitis-Related Genes Across Human Tissues Based on the Summary Mendelian Randomization

**DOI:** 10.3389/fcell.2020.624766

**Published:** 2021-05-06

**Authors:** Jiahui Zhang, Mingai Sun, Yuanyuan Zhao, Guannan Geng, Yang Hu

**Affiliations:** ^1^Department of Stomatology and Dental Hygiene, The Fourth Affiliated Hospital, Harbin Medical University, Harbin, China; ^2^General Hospital of Heilongjiang Province Land Reclamation Bureau, Harbin, China; ^3^Department of Endocrinology, The First Affiliated Hospital of Harbin Medical University, Harbin, China; ^4^School of Life Science and Technology, Harbin Institute of Technology, Harbin, China

**Keywords:** gingivitis, SNPs, genes, summary Mendelian randomization, GWAS, eQTL

## Abstract

Periodontal diseases are among the most frequent inflammatory diseases affecting children and adolescents, which affect the supporting structures of the teeth and lead to tooth loss and contribute to systemic inflammation. Gingivitis is the most common periodontal infection. Gingivitis, which is mainly caused by a substance produced by microbial plaque, systemic disorders, and genetic abnormalities in the host. Identifying gingivitis-related genes across human tissues is not only significant for understanding disease mechanisms but also disease development and clinical diagnosis. The Genome-wide association study (GWAS) a commonly used method to mine disease-related genetic variants. However, due to some factors such as linkage disequilibrium, it is difficult for GWAS to identify genes directly related to the disease. Hence, we constructed a data integration method that uses the Summary Mendelian randomization (SMR) to combine the GWAS with expression quantitative trait locus (eQTL) data to identify gingivitis-related genes. Five eQTL studies from different human tissues and one GWAS studies were referenced in this paper. This study identified several candidates SNPs and genes relate to gingivitis in tissue-specific or cross-tissue. Further, we also analyzed and explained the functions of these genes. The R program for the SMR method has been uploaded to GitHub(https://github.com/hxdde/SMR).

## Introduction

Gingivitis is the most prevalent disease of the periodontium (Oliver et al., 1998) and is commonly known to be a site-specific inflammatory disease caused by the accumulation of dental biofilm (Loe et al., 1965; Theilade et al., 1966; Trombelli et al., 2004). Bacteria in the mouth from gingivitis can easily get into the bloodstream and cause damage to organs. The list of potential problems this bacteria can cause is long. Periodontitis may lead to an increased risk of stroke, heart attack, heart disease, dementia, rheumatoid arthritis, and lung disease (Humphrey et al., 2008; Jamieson et al., 2015). Studies have shown that the prevalence and severity of gingivitis are high (Oh et al., 2002). More than 70% of children older than 7 years old are affected by gingivitis (Stamm, 1986). The clinical symptoms of gingivitis are redness and edema of the gingival tissue, bleeding on provocation, changes in contour and consistency, occurrence of calculus and/or plaque, and lead to tooth loss and contribute to systemic inflammation (No authors listed, 2000; Kinane et al., 2017). Although The pathogenic factors of gingivitis are mainly related to the microbial biofilm of the dental plaque, hormonal fluctuations, drugs, malnutrition, and system disease, the genetic variation, and epigenetic program determine the susceptibility and the regulatory capacity for plaque pathogens (Kinane et al., 2017; Murakami et al., 2018; Zhang S. et al., 2020). Therefore, identify gingivitis-related genes and loci can elucidate disease mechanisms and guide clinical diagnosis and treatment.

Over the first decade of the twenty first century, with the maturity of high-throughput sequencing technology, a large amount of genomic data provides an important platform for researchers to discover abnormal genes related to diseases, understand disease mechanisms, and develop treatment methods (Zhao and Grant, 2011; Zou and Ma, 2019; Wang L. et al., 2019; Cheng et al., 2020). In recent years, several institutions and companies have discovered gingivitis-related pathways and susceptibility genes. For example, polymorphisms in the interleukin-1 gene cluster can influence the severity of gingivitis (Parkhill et al., 2000; Papapanou et al., 2001). Through transcriptome analysis of patients with gingivitis and healthy non-smokers, Demmer et al. (2008) identified 61 differentially expressed genes and function enrichment analysis show these are significantly related to apoptosis, antimicrobial humoral response, antigen presentation, regulation of metabolic processes, signal transduction, and angiogenesis. Kim et al. (2016) also identified 400 up-regulated genes and 62 down-regulated genes which mainly related to defense/immunity protein, receptor, protease, signaling molecules, cytoskeletal, and structural proteins by transcriptome sequencing of gingival biopsies. However, the current research is mainly to identify gingivitis-related genes through biological experiments or simple difference analysis. The major weakness with these study is that it does not incorporate more biological information and describe the disease in a single tissue, which makes it difficult to identify key disease-causing genes from thousands of genes. How to integrate more biological information across multiple tissues has become a research hotspot and challenge (Lewin et al., 2016).

The Genome-wide association study (GWAS) is an observational study that detects the single nucleotide polymorphisms (SNP) of multiple individuals of a specific species to find genetic variations associated with a particular trait (Li et al., 2015; Liu and Jiang, 2016; Jiang et al., 2017; Bush, 2019; Cheng et al., 2019b; Sun et al., 2019). In recent years, there have been many studies that have identified several risk genes associated with gingivitis through the GWAS analysis. The GWAS study of 4,910 European-American adults shows that high IL-1β and IL-6 expression is associated with *IL37* locus variant, which induces more severe periodontal disease (Offenbacher et al., 2018). The genetic variation of *ASIC2* (acid-sensing ionic channel 2) locus, which locates chromosome 17, is significantly associated with severe gingivitis (Zhang et al., 2016). While GSWA can effectively identify disease-related gene loci, there are still many limitations and problems. The GWAS can determine the locus related to the trait or disease instead of directly determining the gene itself. Due to the hypothesis of “disease-common variations,” it is difficult for GWAS to identify rare variants. And these rare variants may be an important role in the disease process (Liu et al., 2016, 2018; Hu et al., 2018). Besides, GWAS only gives statistical conclusions on genetic variants and phenotype, and there is no information on gene function studies. Therefore, GWAS cannot fully reveal the abnormal genetic loci of complex disease. How to accurately identify the genetic variant directly related to the disease and obtain these changed biological functions is a major challenge.

At present, a large number of GWAS studies have found that 80% of genetic variation sites are located in non-coding regions of the genome. At present, a large number of GWAS studies have found that 80% of genetic susceptibility sites are located in non-coding regions of the genome, which indicates that some pathogenic genetic sites may have the ability to regulate gene expression (Hu et al., 2018; Cheng et al., 2019a). The expression quantitative trait loci (eQTL) mapping analysis takes the expression level of genes as quantitative traits and uses traditional OTL methods to identify genetic sites that can regulate gene expression. Traditional QTL methods need to measure the genotype and gene expression level of each individual studied, and then use association analysis (outbred population) or linkage analysis (family or experimental hybrid population) to assess the association between genotype and gene expression level (Rockman and Kruglyak, 2006; Skelly et al., 2009; Albert and Kruglyak, 2015).

The Summary Mendelian Randomization (SMR) is a transcriptome-wide association analysis method that integrates summary-level data from independent GWAS with data from eQTL studies to identify genes whose expression levels are associated with a complex trait (Zhu et al., 2016). The statistical performance of the SMR method will increase with a higher research sample size and it can provide a test to distinguish the causal relationship between the genetic variant and gene expression (Pavlides et al., 2016). Since Zhu et al. (2016) first proposed the SMR method, considerable literature has grown up around the SMR method to predicts complex trait gene targets. Hu et al. (2018) used the SMR method to integrate 2 GWAS datasets and 5 eQTL datasets to identify27 SNPs related to Alzheimer’s disease. Meng et al. (2018) collected the largest GWAS and eQTL meta-analysis data and tested 5,967 genes through the SMR method, which identified two potentially causal genes (ASB16-AS1 and SYN2) associated with bone mineral density. In the study of complex collisions, Porcu et al. (2019) found 36% of genes have no genome-wide significant SNP nearby in previous GWAS by applying the SMR method for 43 human phenotypes and they think that the majority of these loci were missed by GWAS due to power issues. Veturi’s research also believes that the SMR method has excellent capabilities under the assumption of causality (Veturi and Ritchie, 2018; Cheng, 2019). Hence to obtain more accurate results, we used the SMR method to integrate GWAS and eQTL studies to identify genes related to gingivitis.

## Materials and Methods

### Data Collection

Reliable data is the key to further analysis (Liang et al., 2017; Zhang et al., 2017). We downloaded one GWAS data related to gingivitis from the GWAS Catalog database (The NHGRI-EBI Catalog of human genome-wide association studies^1^). Five eQTL studies data, which are five different tissues of patients with gingivitis, are downloaded from the GTEx database (Genotype-Tissue Expression^2^). The detailed data information has been shown in Table 1.

**TABLE 1:** The number of SNP-Gene pairs related to gingivitis in each tissue.

GWAS	eQTL	Number of SNP	Number of gene
Gingivitis and periodontal diseases	Artery_Tibial	4	5
Gingivitis and periodontal diseases	Blood	5	6
Gingivitis and periodontal diseases	Cells_Cultured_fibroblasts	5	5
Gingivitis and periodontal diseases	Nerve_Tibial	3	4
Gingivitis and periodontal diseases	Skin_Sun_Exposed_Lower_leg	4	6

### The Summary Mendelian Randomization Method

Some biological experiments have found that if a genetic variant affects the expression level of a gene, then the gene will have different expression levels among samples who carry different genotypes of the genetic variant (Weiss et al., 2008; McCarthy et al., 2009). In addition, if the gene can also affect the phenotype, the phenotype will be different in different genotypes (Golzio et al., 2012). This process is very similar to the theory of Mendelian randomization (MR) (Katan, 1986; Smith and Ebrahim, 2003). However, the current sample size of phenotype, SNP, and gene expression data cannot meet the needs of MR analysis. For this, we use the SMR method which can integrate summary-level data from independent GWAS with data from eQTL studies to identify genes whose expression levels are associated with a disease phenotype because of pleiotropy (Zhu et al., 2016).

**FIGURE 1:**
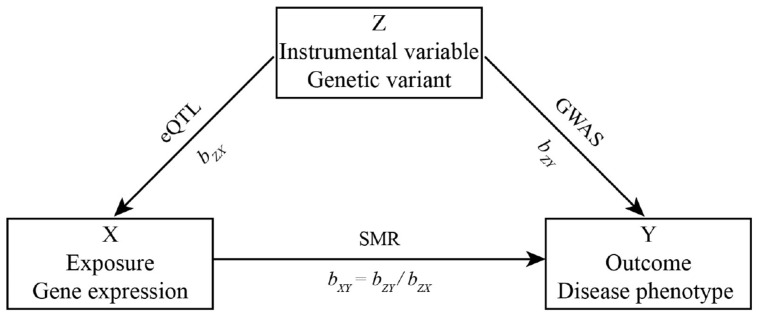
Workflow.

Herein, we let Y be a disease phenotype (outcome), X be gene expression level (exposure), and Z be a genetic variant (instrumental variable). Then, the effect of gene expression on disease phenotype *b*_*XY*_ is *b_*XY*_* = *b*_*ZY*_/*b*_*ZX*_, where *b*_*ZY*_ is the effect of the SNP effect on disease phenotype, *b*_*ZX*_ is the effect of the SNP effect on gene expression. The workflow is shown in Figure 1.

## Results

**FIGURE 2:**
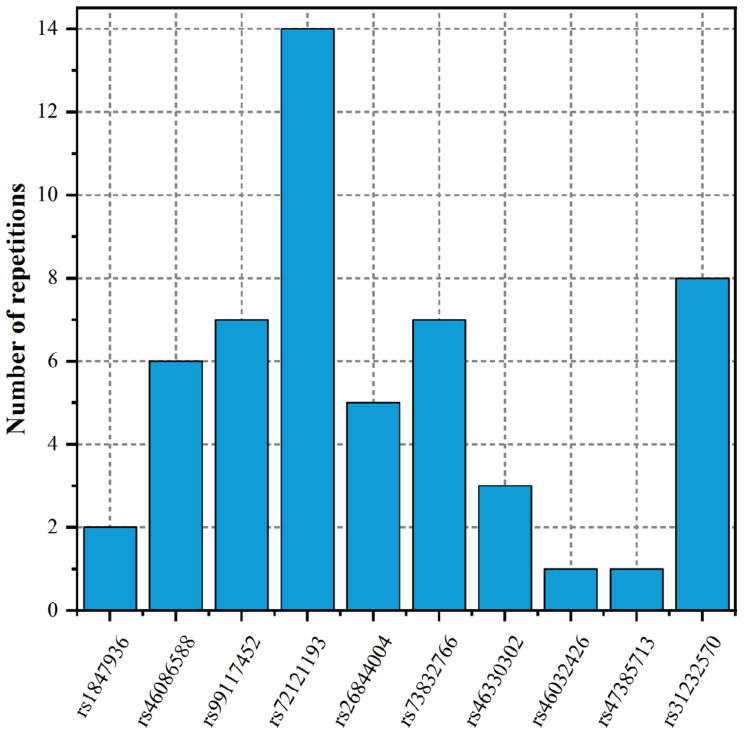
The number of repetitions of SNP-Gene pairs.

To fully elucidate the gene abnormality across the tissues of patients with gingivitis, we downloaded 5 eQTL data from five different organizations (artery, blood, fibroblast, nerve, and skin) and 1 GWAS data (Table 1). The SMR method was used to integrate these data sets to obtain 5 experimental results. For these 5 experiments, we identified a total of 26 SNP-Gene pairs that are significantly associated with gingivitis (Table 2). In the eQTL data, multiple probes can label one SNP, which leads to one SNP that can be repeatedly selected to be associated with gingivitis in 5 experiments. Therefore, we counted the number of times a significant SNP was screened. Looking at Figure 2, in 5 experiments, most SNPs were selected more than four times and only two SNPs were selected once. This result indicates that SNP can be accurately selected in our method.

**TABLE 2:** Information table of SNP-Gene pairs selected by different tissue.

Index	SNP	GENE	*P*-value	TISSUE
1	rs1847936	MYT1L	2.29E-06	Artery
2	rs46086588	FYCO1	2.83E-07	Artery
3	rs99117452	ADH6	2.74E-09	Artery
4	rs72121193	FAM86C1	2.50E-14	Artery
5	rs72121193	ALG1L9P	2.33E-06	Artery
6	rs46086588	FYCO1	2.37E-07	Skin
7	rs26844004	RP11-293A21.1	5.45E-07	Skin
8	rs72121193	FAM86C1	2.73E-16	Skin
9	rs72121193	ALG1L9P	2.04E-10	Skin
10	rs72121193	ZNF705E	1.15E-09	Skin
11	rs73832766	MRPL48	2.48E-06	Skin
12	rs46086588	FYCO1	7.30E-08	Neuro
13	rs99117452	ADH5	1.64E-06	Neuro
14	rs72121193	FAM86C1	9.32E-07	Neuro
15	rs72121193	ALG1L9P	1.76E-07	Neuro
16	rs46086588	FYCO1	3.90E-07	Fibroblast
17	rs46330302	CCR1	1.17E-10	Fibroblast
18	rs26844004	RP11-293A21.1	2.29E-06	Fibroblast
19	rs99117452	ADH4	2.07E-07	Fibroblast
20	rs46032426	CITF22-92A6.2	6.02E-07	Fibroblast
21	rs26844004	RP11-293A21.1	2.15E-06	Blood
22	rs99117452	ADH5	2.59E-12	Blood
23	rs72121193	FAM86C1	4.22E-15	Blood
24	rs72121193	NUMA1	4.20E-08	Blood
25	rs47385713	RP11-493L12.6	3.03E-06	Blood
26	rs31232570	EVI2A	5.98E-08	Blood

### Functional Analysis of Gingivitis-Related Genes

**FIGURE 3:**
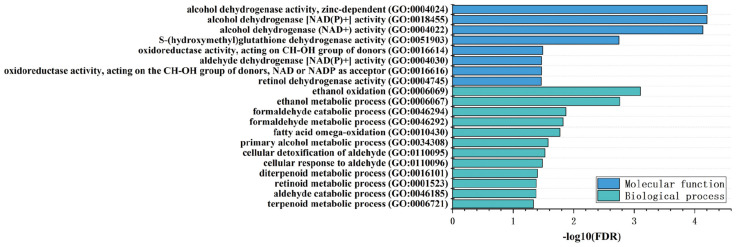
A bar graph of GO function enrichment for gingivitis-related genes.

For the gingivitis-related genes identified by the SMR method, the GO database was used for functional annotation. As shown in Figure 3, these genes are significantly annotated into 12 biological processes and 8 molecular functions.

The top two biological functions are all related to formaldehyde metabolism. A considerable amount of literature has been published on formaldehyde may be a causative factor of gingivitis. The study by Wantke et al. (2000) found that exposure to formaldehyde can induce the production of specific IgE and the research subjects have symptoms such as gingival bleeding, oral or pharyngeal itch. Tokar et al. (2020) test on the oral condition of woodworkers who have long-term contact with formaldehyde showed that exposure to formaldehyde has significant side effects on periodontal diseases such as gingivitis. In addition, there are six biological processes related to the metabolism of ethanol and aldehyde. Several lines of evidence suggest that ethanol and aldehyde both ethanol and aldehyde can damage the oral cavity and induce gingivitis and periodontal disease (Barczynski et al., 1987; Dong et al., 1996; Wyganowska-Świątkowska et al., 2018) and in severe cases, ethanol can even cause oral cancer (Calderón-Montaño et al., 2018). Both alcohols and aldehydes belong to the oxygen-containing derivatives of terpenoids. In our results, the two biological processes related to the terpenoids are also abnormal. Therefore, we infer that the metabolic disorders of alcohol and aldehydes in patients with gingivitis may be related to the occurrence and severity of gingivitis.

The remaining two biological processes are fatty acid omega-oxidation and retinoid metabolic process. Abnormal function of fatty acid omega-oxidation will hinder the metabolism and absorption of fatty acids. However, studies have found that some fatty acids have anti-inflammatory and antimicrobial effects to treat gingivitis (Peedikayil et al., 2015; Woelber et al., 2019). Vitamin A and its analogs have many physiological functions such as promoting growth and reproduction, maintaining bones, epithelial tissue, vision, and normal secretion of the mucosal epithelium (Chapman, 2012). When vitamin A is deficient, the mucosal barriers caused by infection cannot be repaired and the innate immunity is destroyed. At the same time, vitamin A deficiency can also reduce the adaptive immune response mediated by Th2 cells (Stephensen, 2001). Studies have found that the damage to mucosal epithelial regeneration and changes in immune function caused by vitamin A deficiency is important to the occurrence and recovery of periodontal diseases (Cutress et al., 1976; Dommisch et al., 2018). Currently, retinoid medication has been used for the treatment of gingivitis and periodontal disease (Lundgren et al., 1996; Epstein and Gorsky, 1999).

**FIGURE 4:**
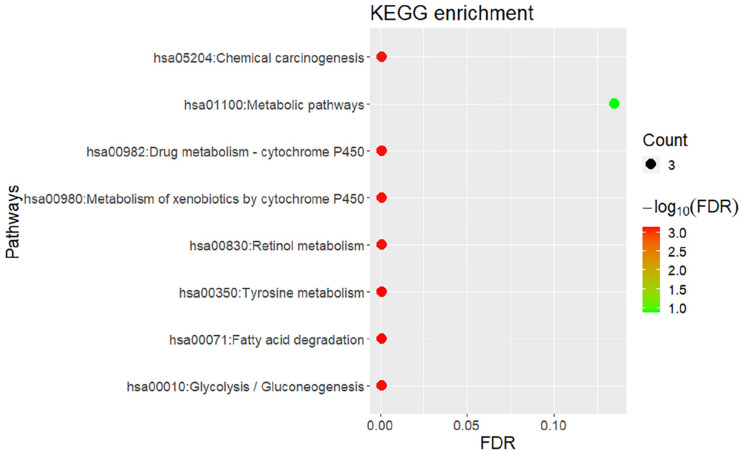
Bubble chart of KEGG pathway enrichment for gingivitis-related genes.

Similar to the enrichment results of biological processes, KEGG pathway enrichment results also indicate that gingivitis-related genes are mainly involved in tyrosine metabolism, fatty acid degradation, retinol metabolism and Glycolysis/Gluconeogenesis, etc. (Figure 4). The molecular functions of gingivitis-related genes are mainly related to the activity of S-(hydroxymethyl)glutathione dehydrogenase, alcohol dehydrogenase, retinol dehydrogenase, and oxidoreductase (Figure 3). This indicates that these biological enzymes play an important role in gingivitis and may be potential therapeutic targets.

### Tissue Related Genes in Gingivitis

**FIGURE 5:**
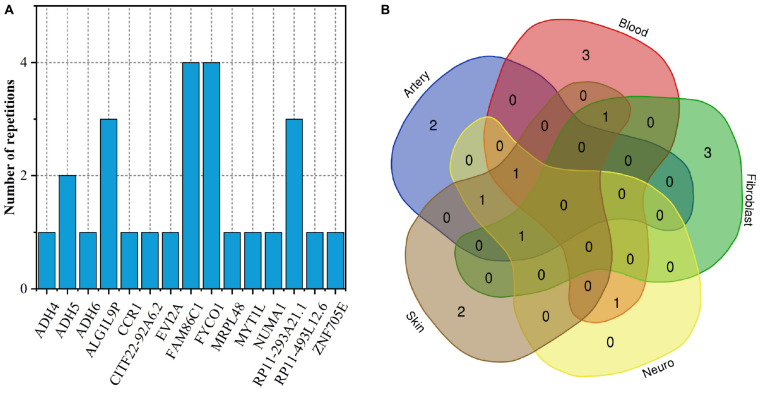
**(A)** The number of duplicate genes in five tissues; **(B)** Venn diagram of gene distribution in tissues.

As shown in Figure 5A, only two genes *FAM86C1* and *FYCO1* occur simultaneously in 4 tissues. They are related to protein methylation, metabolism of proteins and inflammation, the microtubule transport of autophagosomes, respectively (Cloutier et al., 2013; Cheng et al., 2016). The Venn diagram of the distribution of gingivitis-related genes in five tissues shows that the expression of gingivitis-related genes has obvious tissue specificity (Figure 5B). The genes *MYT1L* and *ADH6* are related to arterial tissue; The genes *ZNF705E* and *MRPL48* are related to skin tissues; The genes *CCR1*, *ADH4*, and *CITF22-92A6.2* are related to fibroblast; The genes *NUMA1*, *RP11-493L12.6*, and *EVI2A* are related to blood. The *ADH4* as a member of the alcohol dehydrogenase family metabolizes a wide variety of substrates, including ethanol, retinol, other aliphatic alcohols, hydroxysteroids, and lipid peroxidation products (Tokar et al., 2020). It is worth noting that studies have found that alcohol has an irreversible effect on human gingival fibroblasts (Wyganowska-Świątkowska et al., 2018). In addition, some studies have found that the expression level of *CCR1* tends to change in the gingival fibroblast (Candotto et al., 2019; Lauritano et al., 2019).

**FIGURE 6:**
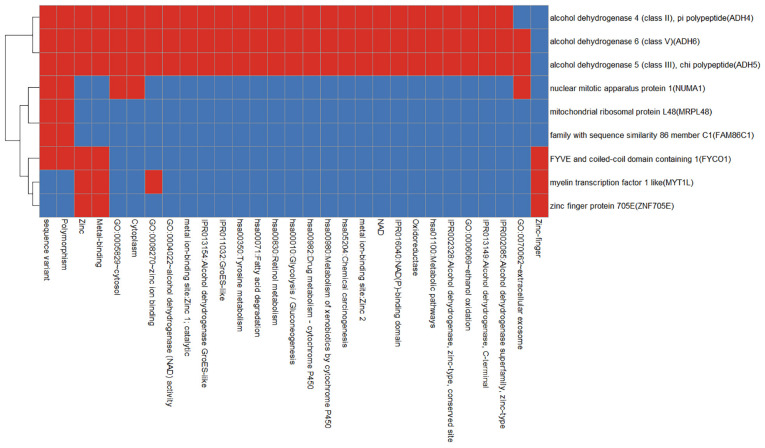
Functional annotation heat map of gingivitis related genes. Red means corresponding gene-term association positively reported and blue means corresponding gene-term association not reported yet.

We use the Database for Annotation, Visualization and Integrated Discovery (DAVID) v6.8 tool^3^ to enrich gingivitis-related genes into the functional set. As shown in Figure 6, apart from the alcohol dehydrogenase family (ADH4, ADH5, and ADH6), other genes have smaller functional overlaps.

### Genes Associated With Gingivitis and Other Diseases

Gingivitis is common chronic inflammation and recent research has suggested it play an important role in the occurrence and development of some systemic diseases (Humphrey et al., 2008; Shah et al., 2010; Hou et al., 2019; Lombardi et al., 2019; Goodson, 2020). We found that some gingivitis-related genes can mediate multiple diseases at the same time.

*MYT1L* (myelin transcription factor 1 like) encodes a member of the zinc finger superfamily of transcription factors. *MYT1L* mutation can lead to intellectual disability and obesity (Blanchet et al., 2017; Loid et al., 2018). Research finds gingivitis and obesity exhibit disease reciprocity and gingivitis is more prevalent in obesity (Dursun et al., 2016; Goodson, 2020). *CCR1* can regulate the transduction of immune signals and affect the recruitment of effector immune cells to the site of inflammation (Foroughi et al., 2016). *CCR1* has an important role in the occurrence of chronic inflammation of gingivitis (Silva et al., 2005). At the same time, it is a target for multiple myeloma and kidney diseases (Ninichuk and Anders, 2005; Vallet and Anderson, 2011). In addition, early diagnosis and treatment of gingivitis can effectively improve the survival expectations of primary liver cancer (Hou et al., 2019). And among genes related to gingivitis, *ADH4* and *FAM86C1* may be potential prognostic and diagnostic markers of liver cancer (Wei et al., 2012; Wang X. et al., 2019).

These abnormal genes in multiple diseases indicate the connection between gingivitis and other systemic diseases, but also predict the mechanism of gingivitis inducing other diseases.

## Conclusion

Gingivitis is a common periodontal disease and inflammation. Gingivitis is mainly caused by a substance produced by microbial plaque, systemic disorders, and genetic abnormalities in the host. Bacteria that infect the human oral can easily get into the bloodstream and cause damage to organs and may lead to systemic disorders and an increased risk of stroke, heart attack, heart disease, dementia, rheumatoid arthritis, and lung disease. Discovering abnormal genes related to gingivitis is important for understanding the disease mechanism, early diagnosis, and treatment of the disease.

Herein, we used the SMR method to integrates summary-level data from independent GWAS with data from eQTL studies to identify gingivitis-related genes. One GWAS dataset and 5 different eQTL datasets from organizations are combined into 5 experiments. In total, we identified 26 SNP-Gene pairs that are related to gingivitis in different tissues. Through GO function enrichment analysis, gingivitis-related genes were enriched into 12 biological processes and 8 molecular functions. A number of studies have confirmed that the functions and genes we discovered are indeed related to the occurrence, development, and treatment of gingivitis and periodontal diseases. These prove the reliability of our results and the accuracy of the method. Besides, we also present gingivitis-related biological enzymes that can be used as potential therapeutic targets and tissue-specific gingivitis-related genes which guide further research on gingivitis on systemic disorders. Machine learning (Zou et al., 2018; Qu et al., 2019; Zou, 2019; Dao et al., 2020; Zhang Z.M. et al., 2020; Zhao et al., 2020) and big data mining will also help in-depth mining biological knowledge.

## Data Availability Statement

The datasets presented in this study can be found in online repositories. The names of the repository/repositories and accession number(s) can be found in the article/supplementary material.

## Author Contributions

JZ and MS wrote the manuscript and did the experiments. YH provided ideas of this work. GG and YZ revised this manuscript and guided how to do experiments. YH supervised this work. All authors contributed to the article and approved the submitted version.

## Conflict of Interest

The authors declare that the research was conducted in the absence of any commercial or financial relationships that could be construed as a potential conflict of interest.

## Footnotes

1 https://www.ebi.ac.uk/gwas/

2 http://commonfund.nih.gov/GTEx/

3 https://david.ncifcrf.gov/home.jsp
